# Rheumatoid Arthritis and the Risk of Bipolar Disorder: A Nationwide Population-Based Study

**DOI:** 10.1371/journal.pone.0107512

**Published:** 2014-09-17

**Authors:** Chih-Chao Hsu, San-Chi Chen, Chia-Jen Liu, Ti Lu, Cheng-Che Shen, Yu-Wen Hu, Chiu-Mei Yeh, Pan-Ming Chen, Tzeng-Ji Chen, Li-Yu Hu

**Affiliations:** 1 Department of Psychiatry, Kaohsiung Veterans General Hospital, Kaohsiung, Taiwan; 2 Division of Hematology and Oncology, Department of Medicine, Taipei Veterans General Hospital, Taipei, Taiwan; 3 Institute of Public Health & School of Medicine, National Yang-Ming University, Taipei, Taiwan; 4 Department of Psychiatry, Chiayi Branch, Taichung Veterans General Hospital, Chiayi, Taiwan; 5 Cancer Center, Taipei Veterans General Hospital, Taipei, Taiwan; 6 Department of Family Medicine, Taipei Veterans General Hospital, Taipei, Taiwan; 7 Department of Psychiatry, Yuanshan Branch, Taipei Veterans General Hospital, Yilan, Taiwan; 8 School of Medicine, National Yang-Ming University, Taipei, Taiwan; University of Missouri-Kansas City, United States of America

## Abstract

**Background:**

Studies have suggested that chronic inflammation plays an essential role in the pathophysiology of both rheumatoid arthritis (RA) and bipolar disorder. The most common clinical features associated with RA are anxiety and depression. The risk of bipolar disorder among patients with RA has not been characterized adequately.

**Objective:**

To determine the association between RA and the subsequent development of bipolar disorder and examine the risk factors for bipolar disorder among patients with RA.

**Methods:**

We identified patients who were diagnosed with RA in the Taiwan National Health Insurance Research Database. A comparison cohort was created by matching patients without RA with those with RA according to age, sex, and comorbidities. The occurrence of bipolar disorder was evaluated in both cohorts.

**Results:**

The RA cohort consisted of 2,570 patients, and the comparison cohort consisted of 2,570 matched control patients without RA. The incidence of bipolar disorder (incidence rate ratio  = 2.13, 95% confidence interval [CI]  = 1.12–4.24, *P* =  .013) was higher among patients with RA than among control patients. Multivariate, matched regression models revealed that asthma (hazard ratio [HR]  = 2.76, 95% CI 1.27–5.96, *P* =  .010), liver cirrhosis (HR  = 3.81, 95% CI  = 1.04–14.02, *P* =  .044), and alcohol use disorders (HR  = 5.29, 95% CI  = 1.71–16.37, *P* =  .004) were independent risk factors for the development of bipolar disorder among patients with RA.

**Conclusion:**

RA might increase the incidence of bipolar disorder development. Based on our data, we suggest that, following RA diagnosis, greater attention be focused on women with asthma, liver cirrhosis, and alcohol use disorder. Prospective clinical studies of the relationship between RA and bipolar disorder are warranted.

## Introduction

Rheumatoid arthritis (RA), an autoimmune disease, impairs patient health and has wider implications regarding health policy and costs. RA primarily affects the joints, but can also affect the entire body, causing systemic symptoms [1]. Several studies have compared patients with RA and healthy controls and have demonstrated that the mechanism involved in the pathogenesis of RA symptoms is related to chronic inflammation and associated with various cytokines [2].

Interest has grown in the psychiatric aspects of autoimmune diseases characterized by chronic inflammation, which might induce psychiatric illnesses through neuroinflammation and neurotransmitter abnormalities. Autoimmune diseases have been proved to be associated with psychiatric disorders, particularly schizophrenia, major depressive disorder, and bipolar disorder [3]. Studies have identified an association between RA and psychiatric disorders, particularly depressive disorder and schizophrenia [4]–[6]. However, the existence of this association remains uncertain. Moreover, few studies have investigated the association between RA and bipolar disorder.

Studies have shown that numerous cytokines circulating in plasma might impair the function of the blood-brain barrier [7], indicating that peripheral inflammation is associated with the upregulation of central nervous system (CNS) inflammation. Several studies have shown that chronic inflammation plays a vital role in the pathophysiology of common mental disorders [8], including bipolar disorder [9]. Therefore, we hypothesized that a history of RA increases the risk of the subsequent onset of bipolar disorder.

To test our hypothesis, we designed a nationwide population-based study to investigate the incidence of bipolar disorder among patients with RA.

## Patients and Methods

### Data Sources

The Taiwan National Health Insurance (NHI) program offers comprehensive, universal health insurance to all residents of Taiwan. The NHI program covers more than 96% of the residents of Taiwan and is contracted with 99% of all hospitals and clinics in Taiwan [10]. The program provides coverage for outpatient, inpatient, emergency, and traditional Chinese medicine services as well as prescription drugs. Multiple NHI databases, including NHI enrollment files, claims data, and a prescription drug registry, are managed and publicly released by the National Health Research Institutes (NHRI) of Taiwan. The Institutional Review Board of Taipei Veterans General Hospital approved this study (2013-03-035AC). Written consent from the study patients was not obtained because the NHI dataset consists of deidentified secondary data used for research purposes; the Institutional Review Board of Taipei Veterans General Hospital issued a formal written waiver for the need for consent. The National Health Insurance Research Database (NHIRD) is publicly released by the NHRI for research purposes only. Although confidentiality is guaranteed according to the data regulations of the NHRI, studies using this database still must be approved by the ethics committee. Detailed information on data requests is provided on the NHRI Web site (http://nhird.nhri.org.tw). Comments, problems, or requests regarding data application can be sent to the following NHRI email address: nhird@nhri.org.tw.

### Study Design and Patients

We conducted a retrospective cohort study of patients who were newly diagnosed with RA between January 1, 2000, and December 31, 2010. We identified RA cases in the Taiwan National Health Insurance Research Database (NHIRD) based on International Classification of Diseases, Ninth Revision, Clinical Modification (ICD-9-CM) Code 714.0 and included only patients with RA who received a catastrophic illness certificate. The NHI program has specified 30 categories of catastrophic illness (e.g., cancers, hemophilia, autoimmune diseases including RA, and chronic renal failure). Eligible patients can apply for catastrophic illness certificates, and if approved, they are exempted from copayment of related medical costs. In this study, we relied on physicians' ability to make accurate diagnoses. However, RA diagnosis involves multiple criteria and ruling out diseases with RA-like symptoms. The possibility that RA diagnoses were misclassified was a concern. The issuance of certificates is validated based on a careful review of medical records, laboratory studies, and imaging studies conducted by at least 2 specialists. Therefore, we included only patients with RA who received a catastrophic illness certificate to ensure that RA diagnoses were valid. Several studies that have examined the epidemiology of systemic autoimmune diseases, particularly RA, by using the NHIRD have been published [11], [12].

Patients with bipolar disorder between January 1, 2000 and December 31, 2010 were identified based on the ICD-9-CM codes for bipolar disorder (296.0X, 296.1X, 296.4X, 296.5X, 296.6X, 296.7X, 296.80, or 296.89). However, to ensure that the bipolar disorder diagnoses in the database were reliable, we collected data on prescriptions of psychotropic agents for these patients. The role of medication in bipolar disorder treatment is firmly established. Therefore, we excluded patients who were diagnosed with bipolar disorder (according to ICD-9-CM codes) but did not receive any medication. We collected information on the use of drugs approved by the Food and Drug Administration of Taiwan for treating one (or more) phases of bipolar disorder, including acute mania/mixed episodes, bipolar depression, and bipolar maintenance. In addition to mood stabilizers, atypical antipsychotics such as aripiprazole, olanzapine, quetiapine, risperidone, and ziprasidone were included. These drugs were classified according to the World Health Organization Anatomical Therapeutic Chemical classification. Only patients who were prescribed these drugs for at least one month were included in our study. Furthermore, patients with mood disorders resulting from a general medical condition (ICD-9-CM Code 293.83) and patients with a history of mood disorders before the enrollment date were excluded from our study.

For each patient with RA in the NHIRD, one patient without RA matched according to age, sex, comorbidities [13], and enrollment date was selected. Although numerous studies have identified several comorbidities as risk factors for RA, based on the inflammation hypothesis proposed in this study, other inflammation-associated comorbidities were considered potential confounders. The exclusion criteria applied to the case cohort were applied to the matched comparison cohort. Both the patients with RA and comparison patients were followed until the development of bipolar disorder, death, or the end of the study period.

### Statistical Analysis

The diagnosis of bipolar disorder served as the primary dependent variable. We calculated the bipolar disorder incidence rates (per 10 000 person-y) and the incidence rate ratios (IRRs). The study groups were compared using the χ^2^ test for categorical variables. The Kaplan-Meier method was used to estimate the cumulative incidence of bipolar disorders, and a Cox proportional hazard model was used to identify risk factors for bipolar disorder in patients with RA. The qualifying criterion for inclusion in the multivariate analysis was a result in the univariate analysis with a P value of less than .1. The Perl programming language (Version 5.12.2) was used to extract data from the databases. Microsoft SQL Server 2005 (Microsoft Corp., Redmond, WA, USA) was used for data linkage, processing, and control sampling. The SPSS, Version 19.0 for Windows (IBM, Armonk, NY, USA) and the SAS, Version 9.2 (SAS Institute, Cary, NC, USA) computer software programs were used to perform all statistical analyses. Comparison results with a P value less than .05 were considered statistically significant.

## Results

### Patient Characteristics

Table 1 shows demographic and comorbidity data on the patients with RA and control patients. The median age of the patients was 51 years (interquartile range  = 41–60 y). The majority of patients in both cohorts were women (75.1%). Diabetes mellitus, chronic obstructive pulmonary disease, asthma, liver cirrhosis, alcohol use disorder, malignancy, chronic kidney disease, cerebral vascular disease, dyslipidemia, hypertension, and coronary artery disease were the most common comorbidities. No statistically significant differences were observed in baseline comorbidity data between the study groups.

**Table 1:** Baseline characteristics of patients with rheumatoid arthritis (RA) and the matched cohort.

Characteristics		Patients with RA n = 2,570 (%)		Matched cohort n = 2,570 (%)		*p* value
Median age, years (interquartile range)		51(41–60)		51(41–60)		
Age, years						
	≥50	1,358	52.8	1,358	52.8	1.000
	<50	1,212	47.2	1,212	47.2	
Sex						
	Male	639	24.9	639	24.9	1.000
	Female	1,931	75.1	1,931	75.1	
Comorbidities						
	Hypertension	790	30.7	793	30.9	1.000
	Dyslipidemia	739	28.8	739	28.8	0.157
	COPD	582	22.6	583	22.7	0.973
	Diabetes mellitus	497	19.3	497	19.3	1.000
	Asthma	408	15.9	406	15.8	0.939
	Chronic kidney disease	329	12.8	329	12.8	0.235
	Cerebrovascular disease	310	12.1	309	12.1	1.000
	Alcohol use disorder	65	2.5	53	2.1	<0.001
	Liver cirrhosis	64	2.5	47	1.8	0.103
	Malignancies	58	2.3	46	2.1	0.264
	Coronary artery disease	27	1.1	24	0.9	0.928
Median follow-up years (interquartile range)		6.1(3.4–8.6)		6.1(3.4–8.6)		0.673

COPD, chronic obstructive pulmonary disease.

### Incidence of Bipolar Disorder

**Figure 1:**
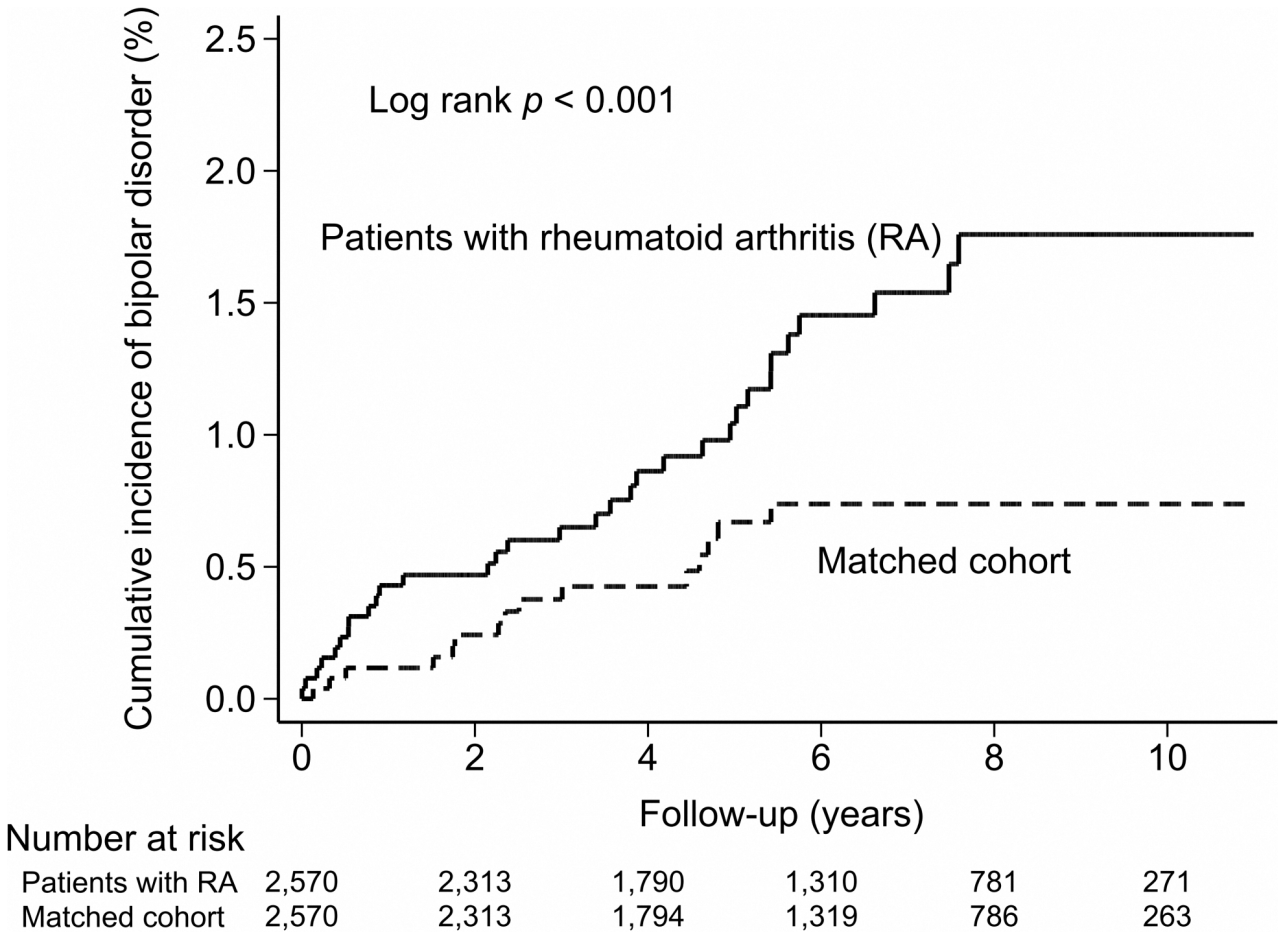
Cumulative incidence of bipolar disorder among patients with rheumatoid arthritis (RA) and the matched cohort.

The cumulative incidence rates of bipolar disorder are shown in Figure 1. As shown in Table 2, the risk of developing bipolar disorder was significantly higher among patients with RA than among patients in the matched cohort (IRR  = 2.13; 95% confidence interval [CI]  = 1.12–4.24; *P* =  .013). After stratifying patients according to age and sex, we observed that patients with RA under 50 years old exhibited an increased incidence of subsequent bipolar disorder (IRR  = 2.26; 95% CI  = 0.93–6.00; *P* = .051), even when no obvious statistical difference was observed. Moreover, women with RA exhibited a higher risk of developing subsequent bipolar disorder than did men (IRR  = 2.25; 95% CI  = 1.10–4.87; *P* =  .017). Furthermore, we stratified patients according to follow-up duration and observed that the risk of subsequent bipolar disorder was significantly higher in the first year (IRR  = 3.67; 95% CI  = 0.97–20.5; *P* =  .035) and more than 5 years (IRR  = 8.99; 95% CI  = 1.25–394.20; *P* =  .012) after RA diagnosis. Overall, our study indicated that the incidence of the development of bipolar disorder following RA diagnosis was 20.7 per 10 000 person-years.

**Table 2:** Incidence of bipolar disorder among patients with rheumatoid arthritis (RA) and the matched cohort.

		Patients with RA		Matched cohort			
		No. of bipolar disorder	Per 10,000 person-years	No. of bipolar disorder	Per 10,000 person-years	Risk ratio (95% CI)	*p* value
Total		32	20.7	15	9.7	2.13(1.12–4.24)	0.013
Age							
	≥50	14	18.5	7	9.3	1.99(0.75–5.84)	0.136
	<50	18	22.8	8	10.1	2.26(0.93–6.00)	0.051
Sex							
	Male	5	13.0	3	7.8	1.67(0.33–10.76)	0.505
	Female	27	23.2	12	10.3	2.25(1.10–4.87)	0.017
Follow-up							
	0–1 y	11	43.1	3	11.7	3.67(0.97–20.50)	0.035
	1–5 y	12	14.6	11	13.4	1.09(0.44–2.73)	0.839
	≥5 y	9	19.0	1	2.1	8.99(1.25–394.20)	0.012

CI, confidence interval.

### Risks Factors for Bipolar Disorder Among Patients with Rheumatoid Arthritis

The results of the univariate and multivariate analyses revealed that asthma (hazard ratio [HR]  = 2.76; 95% CI  = 1.27–5.96; *P* =  .010), liver cirrhosis (HR  = 3.81; 95% CI  = 1.04–14.02; *P* =  .044), alcohol use disorders (HR  = 5.29; 95% CI  = 1.71–16.37; *P* =  .004), and substance abuse (HR  = 37.42; 95% CI  = 10.31–135.82; *P*< .001) were independent risk factors for the development of bipolar disorder among patients with RA (Table 3).

**Table 3:** Risk factors for bipolar disorder among patients with rheumatoid arthritis.

Variables		Univariate analysis		Multivariate analysis^a^	
		HR (95% CI)	*p* value	HR (95% CI)	*p* value
Age ≥50 years		1.28(0.63–2.57)	0.493		
Female		1.77(0.68–4.58)	0.243		
Comorbidities					
	Hypertension	1.17(0.55–2.48)	0.680		
	Dyslipidemia	0.66(0.27–1.60)	0.356		
	COPD	1.79(0.85–3.79)	0.127		
	Diabetes mellitus	1.26(0.55–2.92)	0.587		
	Asthma	3.12(1.50–6.48)	0.002	2.76(1.27–5.96)	0.010
	Chronic kidney disease	0.50(0.12–2.09)	0.340		
	Cerebrovascular disease	1.91(0.79–4.65)	0.153		
	Alcohol use disorder	9.87(3.78–25.80)	<0.001	5.29(1.71–16.37)	0.004
	Liver cirrhosis	4.87(1.48–16.01)	0.009	3.81(1.04–14.02)	0.044
	Malignancies	1.78(0.24–13.03)	0.572		
	Coronary artery disease	3.43(0.47–25.11)	0.226		

HR, hazard ratio; CI, confidence interval; COPD, chronic obstructive pulmonary disease.

a All factors with *p*<0.1 in univariate analyses and age were included in the Cox multivariate analysis, and age, sex was enter in the Cox multivariate analysis.

## Discussion

This is the first population-based study to examine RA as a risk factor for bipolar disorder by using a matched cohort and a maximal follow-up period of 10 years. The major finding of our study was the discovery of a higher incidence of subsequent bipolar disorder among patients with RA. Furthermore, women with RA had a higher risk of developing subsequent bipolar disorder than did men, and asthma, liver cirrhosis, and alcohol use disorder are potential risk factors for developing bipolar disorder.

This study revealed that patients with RA were at a higher risk of subsequent bipolar disorder. We hypothesized that this risk is linked to the mechanisms of immune dysfunction. Bipolar disorder development after RA might result from the inflammatory process activated by RA. In 2012, Lampa et al [14] investigated the influence of peripheral chronic joint inflammatory disease in RA on CNS inflammation and revealed that the chronic peripheral inflammatory process activated by RA might cause the upregulation of CNS inflammation. Studies have evidenced that chronic and mild inflammation in the periphery are key contributors to the pathophysiology of CNS inflammation in bipolar disorder [15], [16] and that dysregulation of the inflammatory process in the brain triggers and exacerbates atherosclerosis, hypertension, diabetes, obesity, and hyperthyroidism [17]–[20]. Animal models have indicated that peripheral cytokines reach the brain through various mechanisms, including a leaky brain barrier, active transport, activation of endothelial cells, and binding to cytokine receptors [21]. In short, cytokines created through infectious and inflammatory processes play a vital role in mediating the cross-talk between the immune system and the brain and are therefore possible contributors to the development of several psychiatric diseases, including bipolar disorder [22], [23].

Epidemiological studies have indicated that men and women are equally likely to develop bipolar disorder [24]. However, certain studies have revealed that sex steroid hormones play an essential role in immune response; specifically immune-stimulating effects caused by estrogen might trigger the inflammatory process. In other words, women are more vulnerable to immune dysfunction than men [25]–[27]. These findings might explain why women with RA had a higher risk of developing bipolar disorder than did women without RA.

When the patients were stratified according to follow-up duration, the incidence of bipolar disorder was significantly higher in the first year and more than 5 years after RA diagnosis. The first result might have been caused by detection bias; patients with symptoms of bipolar disorder might have been diagnosed shortly after RA diagnosis. The second result was compatible with our inflammation hypothesis regarding the association between RA and bipolar disorder. In contrast to acute inflammation, chronic inflammation persists for a prolonged period of time. We hypothesized that, in the long term, the chronic inflammatory process becomes a pathophysiology for the development of bipolar disorder [28].

Our analysis revealed that asthma, liver cirrhosis, and alcohol use disorder were independent risk factors associated with subsequent bipolar disorder in patients with RA. Evidence has indicated that chronic inflammatory processes in asthma and liver cirrhosis, like the pathophysiology of RA, involve cytokine interactions [29]–[31], and this combined and augmented chronic inflammatory effect might subsequently induce bipolar disorder. Moreover, cytokines that potentially cause depression and anxiety in liver cirrhosis [32] might also be associated with the development of bipolar disorder [33]. In addition, alcohol use disorder was associated with subsequent bipolar disorder in patients with RA. Several studies have indicated that alcohol consumption is associated with a lower incidence of disease and an improved quality of life among female patients with RA [34], [35]. However, other studies have revealed that alcohol use disorders and bipolar disorder share certain genetic characteristics and are similar according to neuroimaging and biochemical findings [36], [37]. Thus, alcohol use disorder might be an independent risk factor for the development of bipolar disorder among patients with RA.

Our study is the first retrospective study to examine RA as a risk factor for the development of bipolar disorder. A matched cohort study design comprising a population-based cohort of patients with RA and adequate controls for comorbidity constitute the strengths of our study. However, several limitations that are inherent to the use of claims databases should be considered. First, the diagnosis of RA in the NHIRD was based on the ICD-9-CM code and the issuance of a catastrophic illness certificate. Thus, the severity of RA as a risk factor for subsequent bipolar disorder was not explored. Second, the causal relationship was assessed mainly according to the chronological order in which the 2 conditions were diagnosed. However, both conditions might require long-term treatment, and the possibility that bipolar disorder causes RA cannot be excluded entirely. Third, information was unavailable on several demographic variables such as socioeconomic status, lifestyle, and family history, which might have provided useful information regarding factors that are potentially associated with RA and bipolar disorder [38], [39]. Finally, this epidemiologic study was based on an observational design rather than an experimental design. Data on the relationship between the exacerbation of RA and the severity of bipolar disorder in patients with RA were unavailable. Therefore, the direction of causality in the association between the aforementioned possible risk factors and bipolar disorder development among patients with RA could not be determined.

In conclusion, this study revealed that RA increases the incidence of bipolar disorder development, suggesting that the RA-related inflammatory process is associated with increased expression of neuropsychiatric disturbances. Based on our data, we suggest that greater attention be focused on women, particularly those with asthma, liver cirrhosis, and alcohol use disorder. Additional prospective clinical studies on the relationship between RA and bipolar disorder are warranted.
